# Experiences of caregivers of patients with noncancer diseases readmitted to an emergency department at the end of life

**DOI:** 10.1186/s12904-024-01596-z

**Published:** 2024-11-15

**Authors:** Jose Amado-Tineo, Teodoro Oscanoa-Espinoza, Rudi Loli-Ponce, Marvin Omar Delgado-Guay

**Affiliations:** 1 Universidad Nacional Mayor de San Marcos, Lima, Peru; 2 Hospital Rebagliati-EsSalud, Lima, Peru; 3 Department of Palliative, Rehabilitation and Integrative Medicine, Unit 1414, The University of Texas MD Anderson Cancer Center, 1515 Holcombe Boulevard, Houston, TX 77030 USA

**Keywords:** End of life, Chronic disease, Emergency department, Life change events, Caregiving

## Abstract

**Background:**

When there is limited access to primary care or end-of-life services for patients with chronic diseases, caregivers often need to bring their loved ones to emergency departments (EDs) to solve or control distressing physical and psychosocial-spiritual problems. There is limited literature about the experiences of primary caregivers of patients with nononcologic chronic diseases who are at the end of life and are evaluated in EDs in Latin America.

**Methods:**

We conducted in-depth interviews with primary caregivers of adult patients with advanced and terminal chronic nononcologic diseases who were evaluated in the ED of a hospital in Lima, Peru. This qualitative study employed a phenomenological approach. Themes, categories, codes, and quotes were analyzed using ATLAS.ti 9.1.4.

**Results:**

Twelve primary caregivers, aged 38 to 76 years old, mostly female immediate family members (daughter or wife), participated. They described their experiences in the ED, including feelings of despair and anguish due to prolonged waiting times, insufficient resources, incomplete information regarding the patient’s problems, and “insensitive” treatment by the staff. Some also expressed gratitude for “saving patient’s lives.” They also experienced deficiencies in home care follow-up and patient transfers, which worsened during the COVID-19 pandemic; many times they felt that “they were ignored.” When caring for patients at home, caregivers felt sad, helpless, and frustrated as they observed patients’ progressive deterioration. As patients approached death, caregivers expressed that they tried to “give them all the love” and to have them present for as long as possible, although at the same time caregivers did not want patients to continue to suffer and hoped for “a better place” after this life. Caregivers found their faith to be a source of strength as they continued to care for and be with their loved one until the end.

**Conclusion:**

Caregivers reported “traumatic” and “shocking” experiences during ED care, as well as conflict between wanting the patient’s suffering to end and wanting to prolong their lives. They also expressed feelings of gratitude, resignation, love, faith, and hope.

**Supplementary Information:**

The online version contains supplementary material available at 10.1186/s12904-024-01596-z.

## Background

Advances in medicine have been producing an epidemiologic transition in human health, with longer life expectancy but also increased rates of comorbidities and the need for better related health care [1]. Nononcologic chronic diseases, such as dementia, cerebral vascular disease, cardiac and respiratory diseases, and renal or hepatic insufficiency, are becoming more frequent and reaching more advanced and irreversible stages in which patients develop multiple symptoms and become functionally dependent on caregivers. These events have profound emotional impacts for patients, families, and health care teams. In addition, these diseases can be even more resource-intensive than advanced-stage cancer [2–6].

A person’s experiences encompass the situations, realities, and understandings they have, within their physical, psychological, spiritual, and social context, at various points in their existence. Another way to define experience is as the objectification of thoughts about reality that each individual elaborates. Experience is specific to each person; for example, the death of a family member is experienced differently by different individuals [7–9]. Death is part of the biological process of every person, however, over the last century people have increasingly died in hospitals, under aggressive end-of-life care and away from their families, often experiencing uncertainty and suffering [10].

Caregivers attend to the physical and emotional needs of a sick person; the primary or main caregiver is usually a spouse, child, close relative, or significant other of the sick person. Caregivers fulfill three main functions: responsibility for the care itself, participation in decision-making processes, and providing support to and solidarity with the sick person [11].

Health care systems must adapt to the needs of the populations they serve. Having an aging population growing faster than Europe and North America, Latin America faces a population with increasing burden of chronic illness and a health care system suffering from several shortcomings and significant inequalities [12]. In the absence of access to primary or home care services for chronically ill patients, caregivers often must seek help from emergency services for both acute and chronic health problems. However, the emergency department (ED) is intended to provide acute interventions to preserve the lives of patients with severe acute pathologies and to avoid disabling sequelae. The ED takes a curative and interventionist approach, but this approach does not adequately meet the needs of patients living with chronic diseases and their caregivers [13, 14].

Two qualitative systematic reviews identified the need for better palliative care in the emergency services of high-income countries in Europe, North America, and Australia, highlighting a lack of recognition of palliative care needs, problems in the palliative care system, and staff training [15, 16]. Additional crucial factors suggesting a need for palliative care in EDs are the frequent overcrowding of EDs of highly specialized hospitals, prolonged hospital stays, and frequent readmissions [17, 18].

The end of life involves complex situations for everyone involved. Experiences of one’s own or a loved one’s end of life entail complex cognitive-emotive interrelations that depend on the particular reality and on individual experiences [9]. Therefore, studies of the end of life require a more holistic approach and analysis. In this context, the present study sought to describe and understand the experiences of caregivers surrounding the end-of-life situations of their loved ones with nononcologic diseases who were readmitted to the adult ED at a local hospital in Peru.

## Methods

In this qualitative study, we adopted Husserl’s phenomenological method, which considers a “phenomenon” to be any reality or situation [8]. We employed a hermeneutic interpretative approach that aimed to describe and understand caregivers’ experiences in their contexts then reduce these experiences to understand their essential characteristics, common features, and structure. This approach then reflexively identifies objectifiable acts that give experiences meaning [19–21]. The authors developed the interview guide used in this study (Appendix 1). Four a priori categories were proposed to guide the initial interviews: process of caring, ED experience, palliative care, and end of life [15, 22, 23]. Each category has their own subcategories and specific key elements (codes) that makes them unique.

The study was conducted in a referral hospital of the social security system in Lima, Peru, a 1500-bed institution with medical and surgical specialties in outpatient, emergency, and inpatient care. The adult ED attends patients over 14 years of age in an independent infrastructure with fast care and monitoring areas (180 stretchers in total); 160,000 visits per year are reported, of which 13% are admitted for monitoring (in critical and general areas) [24].

Participants were selected by purposive sampling until thematic saturation was reached. Patients were identified in the hospital registry, and primary caregivers were contacted by telephone to inform them about the study and request participation. Primary caregivers over 18 years of age who had cared for the patient for at least 3 months and who agreed to participate were included. Patients had chronic, nononcologic, end-stage disease and more than one admission to the ED. Caregivers of patients whose disease started before the age of 18 years, those referred from another region of the country, and those whose interviews were not completed were excluded.

In-depth interviews were conducted between July and December 2021. Four general questions were posed according to the a priori categories, and then deeper exploration of the topics was conducted. The interview was conducted by a physician trained in palliative care with support from a specialist in qualitative research. Due to the COVID-19 pandemic, participants chose either a face-to-face interview (following biosafety measures) or a virtual interview. Both types of interviews were audio and video recorded. A field journal was also used to record important observations about the interview.

**Fig. 1:**
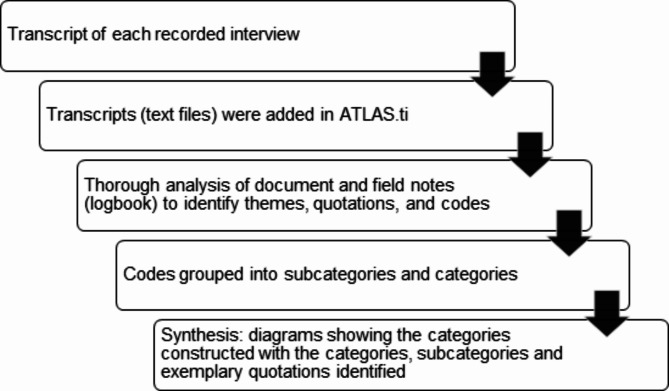
Qualitative data analysis process using a phenomenological approach. Adapted from Mendieta et al., 2015 [20]

Interviews were transcribed, and analysis of themes, categories, codes, and quotes (Fig. 1) **were** performed using the qualitative analysis program ATLAS.ti version 9.1.4 (Berlin, Germany). For the interpretative analysis, an iterative, inductive process of decontextualization and recontextualization (with stages of intentionality, phenomenological reduction, and reconstitution) was followed [20]. A second interview was conducted with each participant to verify the interpretations made.

The study was approved by the Rebagliati Hospital Research Ethics Committee (Note 206-CE-GHNERM-GRPR-ESSALUD-2020). All Participants signed an informed consent document, and the study followed the principles of the Declaration of Helsinki. Data were anonymized after completion of the analysis. The records will be kept in the custody of the lead author on a locked external hard disk for a period of 5 years, after which they will be deleted.

## Results

Twelve caregivers participated; their ages ranged from 38 to 76 years. Most were women, direct relatives (adult daughters) of the patient, married, with higher education level, and actively working. The patients ranged in age from 52 to 93 years, with the most frequent diseases being dementia, renal failure, and heart failure. One face-to-face interview was conducted with a caregiver, and the others were conducted virtually. The interviews ranged in duration from 30 to 55 min. A total of 608 quotes, 44 codes, 11 subcategories, and 4 categories were obtained (Table 1), identifying the experiences in relation to the benefits that the caregiver or patient may have had (Table 2).

**Table 1:** Categories, subcategories, and codes for analysis of primary caregiver end-of-life experiences of patients with non-cancer disease seen in an emergency department in 2021

Categories	Subcategories	Codes
**Considering the emergency department environment**	Infrastructure–System Operations	Arrival at the service, waiting for attention, influences, priority, family visit, environments, inputs
	Hospital Staff	Physicians, nurses, security personnel, clerical, medical report
	Caregiver-Patient	Emotions, treatment received, expectations, treatment received, nasogastric tube, bedsores, accompaniment by the family member
**Considering other aspects of the health system**	Hospitalization area	Staff care (nursing and medical), hospital discharge, ancillary examinations, COVID-19
	Home care	Home visits, primary care physician, COVID-19
	Transfer to the hospital	Distance from home, hospital discharge, who indicates need for emergency care, who moves patient, transfers
	Outpatient care	Outpatient consultations, COVID-19, administrative formalities, virtual support
**Assuming care at home**	With the patient	Patient’s condition, who is caring for patient, care required, treatment received
	With the family	Effect on family life, who takes care of family
**Facing the end of life**	Expectation and quality of life	Quality of life, expectation in the face of death
	Palliative care	What is palliative care, have they received it, the needs of palliative care

**Table 2:** Representative comments for each category and subcategory

Subcategories	Quotes
Considering the emergency department environment	
Infrastructure–System Operations	It was a little bit traumatic… they didn’t even want to listen to me… didn’t even let me say goodbye to my dad. (ESTHER) There is a lot to wait for, too much I would say… and in the cold (PERCY) We waited from nine at night until five in the morning to be evaluated. (JESUS) The environment is very small … there is a lack of stretchers … the staff does not stock up, there are days when the emergency room is very full. (DORIS) There, they saved her life. (FELIPE) They received her well… in the justified time. (DAVID)
Staff attending	There are good doctors and bad doctors, doctors who have an interest in others and others who do not. (FELIPE). The nurses are also others (not all of them), but they don’t have a good treatment either. (ANGEL) Sometimes I wanted them to support me, I started to cry, but they didn’t pay attention to me…they should have a little more humanity. (JESSICA) They answer you in a bad way. (JACKY) The doctor says one thing and the guard says another. (MARY) There are very beautiful nurses … a very good human quality. (FELIPE) He told me, madam pray a lot if you are a believer. (ESTHER) Thank all the health professionals for the work they do every day … the security staff has to comply with the protocols … they have never created false expectations. (DORIS)
Caregiver-Patient	I would have avoided bedsores on my patient if they would have given me permission to come in. (JESSICA) To see her veins not being found…and to see her screaming with pain is horrible. (JACKY) It’s such a terrible anguish… not knowing exactly what your loved one’s condition is. (ESTHER) I was only informed about her condition… but not seeing her for a week, I don’t think it’s good. (PERCY). From the entrance gate, the security people, I have had to juggle to see my mom. (JESSICA) They have taken care of her and they have provided her good attention. (DAVID)
**Considering other aspects of the health system**	
Hospitalization area	The nurses said he is still breathing, she said he has to talk to the doctor. (FELIPE). What do you mean discharged? if there are still things pending, lots of doubts without resolve. (JACKY) I’m going to give her a pass to enter, you can come every day. (JACKY) They have always treated her well, willing to take care of us… and to try to make her feel better. (DORIS)
Home care	They don’t pay attention to us. (ANGEL) Unfortunately, the home service is very bad. (PAOLA) Before the pandemic, they used to come every 10 days, there was a concern. (JESUS)
Transfers to the hospital	I live far away… to get her back… they wouldn’t give me help either. (FELIPE) Ambulance doesn’t come… two, three hours, they take too long, there is no ambulance unit. (PAOLA) They have been timely, very professional, it must be because I live so close to the hospital. (JESÚS).
**Assuming care at home**	
State of the patient	To see her screaming in pain is horrible… She’s not the same person anymore, she’s a crystal. (JACKY) It’s a drastic change from being an independent woman to having to take care of her. (MARY).
With the patient	There is too much care, too much from the beginning, the cleanliness, as I told you, they sent her to me with bedsores. (JESSICA). I was forced to take care of her in my own way … I take care of her as I can, I am not a doctor or a nurse. (PAOLA) We are happy to help because it is very little for all that she has given us. (FELIPE) I entrusted myself to God and I said I have to continue. (JACKY) We fought and we will fight until the end. (JESUS)
With the family	Sometimes family members are unaware of many situations due to lack of experience and we need support, guidance, to be listened to. (JACKY). We are divided between the person who helps us and my brother… there is no problem. (FELIPE). It has left us with a very big emptiness, but we have remained resigned. (DORIS)
**Facing the end of life**	
Expectation and quality of life	I know this moment is going to come… I am resigned, why? Because I’ve seen so much pain in her. (JACKY) If it happens, I would like it to happen at home and that we can say goodbye. (ESTHER) We are getting used to that idea, it is sad, it is irremediable, but we have to accept it… in a dichotomy, we don’t want her to suffer. (JESUS) An end that he did not deserve, but that’s life, diseases do not choose color, race, profession, nothing, unfortunately it touched him. (DORIS). Give him all the love… give him back, even if it’s just a little. (FELIPE) Having a balance between the physical, spiritual and psychological. (CRISTIAN) It comforts us to know that he is in a better place, where there is no pain, no suffering. (DORIS)
Palliative care	I don’t even know what to give him. (JESSICA) It’s just going to calm you down…if I had cancer. (ESTHER) Well palliative, well I don’t know that term. (PERCY) For all types of illness… palliative care to decrease the physical and psychological impact… (DAVID) He has received, requires and will need palliative care until the end. (JESUS)

The names in the table are pseudonyms given to caregivers to protect their identity

### Category 1: experiences in the hospital ED

Participants recounted “very unpleasant” experiences related to inadequate conditions on arrival at the facility, waiting areas for care or reevaluation, exposure to the cold (e.g., in tents with cardboard), lack of adequate furniture (chairs or stretchers), and small or overcrowded environments with many patients and family members waiting, exposing them to potential infections. They also reported incomplete or repeated taking of medical histories (“the doctor who reevaluates starts all over again”) and slow testing procedures and processes (e.g., laboratory tests or imaging). Other caregivers mentioned that “they saved [their patient’s] life there” or that the patient came out in much better condition because they received very good care.

Caregivers were concerned about understaffing; the rotating shifts of the ED personnel made it hard for them to know who was attending the patient. Caregivers also reported that there were some “good professionals and others not so good.” Care quality depended on the time of day; in the early morning, care was slower. Some caregivers described the medical and nursing staff as “spoiled, despots, uncommunicative, tired, insensitive.” Cases of preferential treatment of the ED’s own personnel were also mentioned. These experiences caused suffering to patients and family, who asked for more humane treatment.

The lack or delay of information on the patient’s condition caused discomfort and several complaints from caregivers. Most information was conveyed by telephone, which sometimes was not coordinated with the ED staff, some of whom had an evasive attitude or expressed verbal or nonverbal aggressiveness. Caregivers also reported some satisfactory situations in terms of the information they received virtually and daily on the patient’s progress, in particular care recommendations and spiritual support.

Caregivers mentioned some very upsetting facts, such as finding their patient “strapped to the stretcher,” having catheters (nasogastric or urinary), in insufficiently clean conditions, or with bruises or pressure ulcers/bedsores.

Caregivers also had significant difficulties and limitations in visiting or accompanying their family member, largely because of COVID-19. They expressed feelings of sadness, uncertainty, anguish, helplessness, and resignation; some re-experienced these feelings during the interview and broke into tears. They also experienced significant anguish and stress related to phone calls from the hospital because they feared hearing bad news.

Some caregivers expressed gratitude for being able to visit their patient (with personal protective equipment during the pandemic) and understood the protective measures implemented. They also “felt good” about using some treatments (such as nasogastric tubes) to temporarily help the patient, even though they knew the patient’s prognosis was poor and life expectancy was short.

### Category 2: experiences in other aspects of the health care system

One caregiver reported that the patient received “bad” care in a hospital ward compared to a previous hospitalization. The caregiver explained that physicians had been absent or delayed in the afternoon and evening, that little information had been provided, and that the patient’s treatment had been inadequate. This caregiver also mentioned unplanned and pressured discharges. But another mentioned that their patient was allowed to be accompanied and helped by a caregiver in a calmer environment and that they had received good information as well as kind and sincere treatment from the health care personnel.

Home care was dramatically affected by the COVID-19 pandemic: “it did not solve the patient’s problems,” so caregivers and patients had to go to the hospital ED. The family doctor who used to visit them monthly no longer responded, sometimes they lacked medicines, and they did not receive visits from the rest of the care team, despite multiple phone calls. In addition, procedures took a long time or appointments were long delayed.

Caregivers also reported difficulties in both taking patients to the hospital and returning home. Mobile units (ambulances) for transfers were lacking, often forcing caregivers to look for other services or even transport the patient in private vehicles. Caregivers also noted delays as well as rushed or inadequate handling by transportation personnel. One caregiver expressed his satisfaction with the patient’s transfer, mentioning that this may have been because he lived very close to the hospital.

Because of the pandemic, there were no appointments for outpatient consultations available, and some appointments were only virtual (phone call). Even so, many times calls were not made, or the patient’s problems were not solved. There were delays in administrative procedures and lack of resources for some diagnostic procedures. In some cases, caregivers asked for diapers, commenting that “if the patient or family member paid an economic amount for health insurance, nothing should be paid or purchased separately.”

### Category 3: experiences at the patient’s home

Caregivers described patients’ being in a prolonged state of total dependence (some more than 10 years) and of progressive worsening after each hospitalization (“she has never been the same person again; she is like a crystal”). They expressed sadness and helplessness due to the patient’s physical and emotional problems that they were often not prepared to deal with or alleviate. Too much care was needed at home, and the help of several other people was almost always needed to help in activities such as grooming, feeding, medication administration, position changes, and healing. Many times, caregivers did not know what medications to administer and treated patients “according to what is recommended by non-specialized personnel” (such as other caregivers, relatives, or pharmacists).

Family members’ emotions and family roles were powerfully affected by the patients’ illnesses—younger children due to the parent’s serious illness, older children because they became caregivers for their parents, and spouses because of the need to provide care and support. Home habits and environments had to change, and caregivers described difficulties with their work, studies, and finances. Yet families reorganized and adapted to provide the best care to the patient, with a sense of gratitude for what the patient meant to them and had done for them previously. Caregivers also described disputes or disagreements among family members due to a lack of commitment to the patient’s care and increased burden among the family members caring for the patient.

### Category 4: experiences related to the end of life

Caregivers faced a “dichotomy” at the end of the patient’s life: “I do not want them to die, but neither do I want him/her/them to suffer.” They also expressed resignation before the death of the loved one as well as an understanding that death is part of life and the end of a distressing disease that the patient had suffered for a long time. They expressed wishes that when the end comes, they would be with their loved one at home, where they would be surrounded by their family.

Some caregivers experienced frustration and did not understand “why all this happened to their loved ones” (“if she was good and so young”), even though they knew what the outcome would be and accepted it because of the suffering that their loved one had endured. Caregivers expressed that they found “strength in God to continue caring” for their relative and to continue to “fight until the end.” They expressed the importance of seeking the best quality of life for their loved ones and of balancing the physical, psychological, and spiritual elements/challenges of life. They expressed that they felt satisfied for having cared for their loved ones and were grateful for what they had experienced. Other caregivers stated that they had not been clearly informed about their patient’s prognosis and hoped that they would recover and return to the way they were before.

Several caregivers were unfamiliar with the meaning of palliative care or related it only to cancer. However, they did recognize that their patients needed the symptom control and quality of life that palliative care can provide, but they were not prepared for it, nor did the health care system fully provide it. Trained personnel were needed, and the family needed to be committed to providing the best care possible to their loved ones.

## Discussion

**In this study**, the caregivers of patients with nononcologic chronic disease who are near the end of life and who frequently visit the hospital ED recounted both positive and negative experiences: negative experiences related to the inadequate functioning of the health system, the lack of staff training in palliative care, and deficient resources in infrastructure and supplies, and positive experiences related to receiving specialized care, commitment to the patient, and feelings of gratitude, resignation, and hope. This is a subject on which little has been published in Latin America.

In Latin America, the care of patients with advanced diseases generally falls on a close relative (such as a wife or daughter) and is carried out at home until the end, unlike in high-income countries where there are personnel and institutions dedicated to end-of-life care [23, 25, 26]. This makes the caregiver, who is often afraid of not doing the right thing [10] and has little knowledge of palliative care, seek support in the parts of the health care system that are accessible to them, such as the ED, where their patients often do not receive the best or most appropriate care and experience additional suffering [27–29].

The use of qualitative research and specifically the phenomenological method allows us to approach this subject from a broad and holistic perspective. This method enables an understanding of the human being in all their various aspects and does not limit this understanding to measurable, quantifiable factors. Such quantitative approaches are often insufficient to understand the complex situations of patients and families in the context of terminal illnesses [8, 20].

### Experiences in the ED and the health care system

An important proportion of the patients in our study were older adults, it is reported that this population with multiple comorbidities attends to emergency services with relative frequency, even in situations with low acuity issues. This can be secondary to numerous factors associated not only with the health care system but also to factors related to the non-supported role of the caregivers [30–32].

We found that caregivers often experienced feelings of illegitimacy and inadequate treatment in the ED, as this environment is intended for curative and recuperative treatments for acute events. In patients with chronic diseases, however, the focus of care is on improving quality of life and alleviating discomfort. This discrepancy has also been reported in countries where the health system is highly developed [33, 34], and the need to improve the quality of care with staff training and better resources is widely recognized [15, 33]. Other factors such as stress and work overload among hospital staff also play a role in the quality of care.

Caregivers reported negative experiences regarding communication between the patient, family members, and health care workers, as in other reports on hospitalized older adults in Peru [35] and patients in emergency centers in Italy [33] and the United States [34]. Other negative experiences included moments of feeling out of control or desperation, similar to previous reports [23, 34], as well as concern for the pain and suffering of the patient [36]. Even though the patients whose caregivers were included in this study had advanced chronic diseases, caregivers also expressed uncertainty about their patient’s diagnosis, treatment, and prognosis, similar to reports from high-income countries [25, 34].

Modern health care workers have an eminently technical-scientific role, and ED treatment can seem cold, mysterious, and abrupt. However, the human aspect, with an empathetic, integral approach that includes spiritual support, is a necessary part of good-quality health care [33, 37].

ED infrastructure, resources, and organization in turn involve the entire health care system. Caregivers highlighted problems with their ability to accompany patients and with inadequate environments that lacked privacy and intimacy for the patient or family, important aspects of quality care [33, 38]. Administrative, structural, and operational barriers to health care access [15, 39] exist even in high-income countries, with fragmented care and problems in coordination and communication [34]. However, we were able to identify no publications on such barriers in low-income settings, where this problem is thought to be greater.

Negative feelings related to hospitals, such as associations with danger, isolation, and suffering have also been reported in patients with nononcologic chronic disease, as have positive experiences such as resilience, solidarity, and family support [5, 23, 25]. In the present investigation, appreciation for prompt ED care, resignation, and hope were all identified.

### Experiences at home

The caregivers in our study expressed problems with symptom management at home, in agreement with previous reports [34], due to the complexity of symptoms presented by late-stage chronic diseases, difficulty in administering medications [23, 40], or problems associated with the background disease such as cognitive impairment [22, 41]. Considering the patient as very fragile (“like a crystal”) has been associated with negative stereotypes, especially of elderly patients [42], which may lead to violations of their autonomy.

Both nuclear and extended families are affected by the condition of patients with advanced nononcologic disease, who require extensive care over long periods [5]. In our environment, in contrast to the professionalized system of caregivers available in other countries, the assumption that female family members are obliged to assume caregiving responsibilities persists and is associated with physical, psychological, and even spiritual problems [22, 37]. This problem is not adequately addressed by the health care system, and it should be recognized that more support is needed for the family caregiver than for the professional caregiver [26].

These experiences affect the caregiver’s life, are related to their beliefs [22], and are sometimes even associated with abandonment by the family. In our study, the latter was not frequent because the patients belonged to a social security system; this is a problem that affects more vulnerable populations and those with limited economic resources. For these reasons, it is necessary to prepare the caregivers for this stage; a recent publication of The Lancet Commission mentions that health care personnel should prepare caregivers for all aspects of the patient’s death including communication and spirituality and existential factors, not only for medications, hospitalizations, and emergency care [10].

### Experiences at the end of life specifically

The experiences of patients and their families around death are full of paradoxes [10]; many patients die with expensive hospital care while also not receiving adequate care (which in this case would be quality palliative care). There is also a “dichotomy” faced by caregivers between wanting to “have their patient as long as possible” but at the same time not to prolong their suffering. This further increases caregivers’ experiences of uncertainty [34].

Death is a natural process and is not exclusively a medical or scientific phenomenon. It influences beliefs and spirituality, and studies have even developed a “literacy” model of death [10], that is based on aspects related to the patient, the family environment, and health resources during the end-of-life process. Spirituality (which may or may not be associated with religious belief) has a great influence in the process of facing the end of life, in the sense of resignation experienced by caregivers and family members, and in mourning [22]. Indeed, in Latin American countries, spirituality is considered one of the motivational factors of the act of caring [23].

The experiences of caregivers of relatively young patients who have small children can be shocking; in some cases, this situation brings a family closer together, but in others there is an abrupt separation “to avoid suffering” that may produce greater problems for the children in the future.

### Limitations

The present study was conducted during the COVID-19 pandemic, isolation measures and changes in health care systems, restricted the ability of caregivers to accompany patients in their care. This unique and challenging situation increased the burden and disruption of the healthcare system, the non-availability of homecare services, and the overcrowded and shortage of infrastructure at ED. Although virtual communication was an alternative solution, it was not available in many places [26, 41, 43, 44]. The patients and caregivers included in our study did not represent the entire chronically ill population, and because the hospital where the research was conducted belongs to the social security system, our participants did not include the poorest sector of the population, which does not have adequate access to the health care system, nor were there participants from minority populations. No elderly female caregivers participated, probably due to lack of access.

Although the sample size is small, based on the qualitative approach,this number of participants was sufficient to saturate the categories proposed in the study. Also, our in-depth analysis of this topic allowed us to identify real situations and to raise new avenues for study or solutions to problems.

### Final considerations

Primary caregivers of patients with advanced-stage non-oncologic diseases who were readmitted to the ED generally recounted “traumatic” and “shocking” experiences. Although there were few cases in which caregivers experienced humane and personal treatment, the spiritual sphere seemed forgotten. Caregivers’ positive or negative experiences were related to the number and quality of health personnel interactions, to the functioning and infrastructure of the ED, and to other aspects of the health care system, such as the need for outpatient or home follow-up and palliative care involvement, or the lack of long-stay inpatient facilities. Caregivers also expressed feelings of gratitude, resignation, and hope, and experienced paradoxical feelings of wanting their loved one not to die while also wishing for the end of their suffering.

The evidence reported from this Latin American country shows a reality shared in many areas of the world, where the health care system has large gaps in infrastructure and equipment related to the low resources available, but also to an important aspect in the training and preparation of health care providers, from professional training (with the need for basic knowledge of palliative care) to the inclusion of specialized skills in hospital services with the comprehensive emergency centers. We must also improve the education of the community in general about palliative care services and how to improve the quality of life of this population and support caregivers of patients living with chronic illnesses.

## Electronic supplementary material

Below is the link to the electronic supplementary material.

Supplementary Material 1

## Data Availability

No datasets were generated or analysed during the current study.
