# Supplementary material for: Experiences of caregivers of patients with noncancer diseases readmitted to an emergency department at the end of life
Source: BMC Palliat Care. 2024 Nov 15;23:265. doi: 10.1186/s12904-024-01596-z (PMC11566118; doi:10.1186/s12904-024-01596-z)
Supplement: Supplementary file 1 — Supplementary Material 1 [file 12904_2024_1596_MOESM1_ESM.docx]

**APPENDIX 1**: Interview guide for caregivers of patients with advanced non-cancer diseases regarding emergency department and end-of-life experiences.

**First encounter with the caregiver**

**INTRODUCTION:**

Good day, my name is _ _ _ _ _ _ _ _ _ _ and I am a health care worker from _ _ _ _ _ _ _ _. The purpose of this interview is to collect information about the experiences of caregivers of patients with advanced diseases. For this reason, I will ask some questions that I hope you can answer freely, remembering that all answers are valid; there are no right or wrong answers.

I will make some notes in my notebook and the interview will be recorded so that I can further analyze the information provided. I would like to remind you that as indicated in the informed consent you signed, the data you provide are confidential, will be guarded by me and will only be used in the present investigation.

**GENERAL INFORMATION:**

- How old are you?

- What is your marital status?

- What is your level or degree of education?

- What is your occupation?

- How are you related to the patient?

- How long have you been caring for the patient?

**MAIN ASPECTS OF THE INTERVIEW:**

1. **Aspects related to the process of caring for the patient:**

Guiding question 1: TELL ME: How do you care for your patient at home or place where you live?

Do you receive help caring for the patient? How does caring for the patient affect the family?

2. **Aspects related to the emergency department:**

Guiding question 2: What has been your experience in bringing your patient to the emergency?

How were you cared for in the emergency room? What do you expect from the emergency room?

3. **Aspects related to palliative care:**

Guiding question 3: Do you know what palliative care is, have you heard about it, what do you think?

Do you think your patient needs palliative care?

4. **End-of-life issues**:

Guiding question 4: How do you feel about the possible death of your patient?

How do you perceive the end of life of your family member?

What do you think would be the best care for your patient at the end of life?

**SOCIODEMOGRAPHIC FORM OF THE PATIENT:**

Code: Age: Sex:

Origin: Home or Nursing Home

Diagnosis:

Reason for admission:

Number of admissions to the emergency department in the last 3 months:

INTERVIEW TIME: Start: End:

**Second encounter with the caregiver**:

Date and time:

Do you agree with the data and the interpretation made: YES - NO

Observations:
